# Lysine Residue 185 of Rad1 Is a Topological but Not a Functional Counterpart of Lysine Residue 164 of PCNA

**DOI:** 10.1371/journal.pone.0016669

**Published:** 2011-01-31

**Authors:** Niek Wit, Peter H. L. Krijger, Paul C. M. van den Berk, Heinz Jacobs

**Affiliations:** Division of Immunology, The Netherlands Cancer Institute, Amsterdam, The Netherlands; Tulane University Health Sciences Center, United States of America

## Abstract

Monoubiquitylation of the homotrimeric DNA sliding clamp PCNA at lysine residue 164 (PCNA^K164^) is a highly conserved, DNA damage-inducible process that is mediated by the E2/E3 complex Rad6/Rad18. This ubiquitylation event recruits translesion synthesis (TLS) polymerases capable of replicating across damaged DNA templates. Besides PCNA, the Rad6/Rad18 complex was recently shown in yeast to ubiquitylate also 9-1-1, a heterotrimeric DNA sliding clamp composed of Rad9, Rad1, and Hus1 in a DNA damage-inducible manner. Based on the highly similar crystal structures of PCNA and 9-1-1, K185 of Rad1 (Rad1^K185^) was identified as the only topological equivalent of PCNA^K164^. To investigate a potential role of posttranslational modifications of Rad1^K185^ in DNA damage management, we here generated a mouse model with a conditional deletable *Rad1* ^K185R^ allele. The Rad1^K185^ residue was found to be dispensable for Chk1 activation, DNA damage survival, and class switch recombination of immunoglobulin genes as well as recruitment of TLS polymerases during somatic hypermutation of immunoglobulin genes. Our data indicate that Rad1^K185^ is not a functional counterpart of PCNA^K164^.

## Introduction

Maintaining DNA integrity is crucial to the survival and reproduction of all organisms. As a consequence, elaborate mechanisms have evolved to preserve genetic information. Cells rely on a complex protein network capable of sensing specific DNA damage and triggering adequate responses. Distinct DNA damage checkpoints can delay specific phases of the cell cycle and this extra time window allows a cell to repair or transiently tolerate DNA damage. If the damage is too severe, the system can force the cell to go into senescence or apoptosis [1]. Inappropriate DNA damage management has been associated with a variety of diseases, like cancer and premature ageing [2].

DNA sliding clamps and post-translational modification (PTM) thereof play important roles in DNA replication, recombination, and repair, as well as DNA damage responses (DDR), and DNA damage tolerance (DDT) [3]. The homotrimeric DNA sliding clamp Proliferating Cell Nuclear Antigen (PCNA) encircles the DNA and acts as a critical processivity factor for the replicative polymerases δ and ε. In the presence of stalling DNA lesions, for instance caused by DNA alkylation or UV exposure, prolonged exposure of single-stranded DNA may ultimately lead to the formation of DNA double strand breaks. To prevent the formation of such detrimental secondary lesions, DDT enables DNA replication to be continued. This feature renders DDT as an integral component of the overall cellular response in surviving genotoxic stress [3]. In eukaryotes two DDT pathways are distinguished: translesion synthesis (TLS) and template switching [4]. Both pathways, initially identified as the Rad6 epistasis group, strongly depend on DNA damage-inducible, site-specific ubiquitylation of PCNA at lysine (K) 164 [5]. DNA damage-inducible monoubiquitylation at PCNA^K164^ (PCNA-Ub) is mediated by the E2 conjugase Rad6 and the E3 ligase Rad18 and recruits TLS polymerases via their ubiquitin binding motifs [6], [7], [8], [9]. These TLS polymerases are capable of replicating directly across damaged DNA templates [3]. TLS polymerases have an extended catalytic domain that can fit non-Watson-Crick base pairs, allowing this class of polymerases to synthesize directly across DNA lesions [10]. Simultaneously, the inherent lack of proofread activity renders TLS polymerases error-prone, even in the presence of an intact template. Further K63-linked polyubiquitylation of PCNA-Ub stimulates template switching, which enables stalled replicative polymerases to bypass the damage by switching transiently to the intact template strand of the sister chromatid [4].

Interestingly, affinity maturation of antibodies takes advantage of error-prone TLS polymerases to introduce point mutations at a high rate into the variable region of immunoglobulin genes of B cells, a process known as somatic hypermutation (SHM) [11]. To initiate SHM, the activation-induced cytidine deaminase AID is induced transiently in activated B cells to create uracil residues in the variable region of Ig genes by deaminating cytidines [11], [12]. It is thought that three major pathways can process the U:G mismatch in an error-prone manner. 1) Direct replication of the uracil results in G/C to A/T transitions, as U instructs a template T to DNA polymerases [13], [14]. 2) Excision of the U by the base excision repair protein Ung2, generates a non-instructive abasic site that can be processed by specific TLS polymerases [15]. 3) Alternatively, the U can be recognized as a U:G mismatch by the mismatch recognition complex Msh2-Msh6, resulting in exonuclease 1 (Exo-1) activation, formation of a single-stranded gap, activation of Rad6/18, PCNA-Ub and recruitment of the TLS polymerase η (Pol η) to generate 90% of all A/T mutations around the initial mismatch [16], [17], [18], [19], [20]. Interestingly, TLS polymerases involved in G/C transversions, like Rev1, are not controlled by PCNA-Ub. This suggests that G/C transversions are regulated differently [16], [17]. For instance, this may involve ubiquitylation of the alternative DNA sliding clamp 9-1-1 (see below) [21].

Besides the homotrimeric PCNA DNA sliding clamp, a heterotrimeric DNA sliding clamp exists, Rad9-Rad1-Hus1 (9-1-1), which is evolutionary and structurally highly related to PCNA [22], [23]. While its role as DNA damage sensor in the DDR is well-defined [24], more recent reports revealed a role of 9-1-1 in DDT. The non-catalytic Rev7 subunit of the TLS polymerase ζ a heterodimer of Rev3 and Rev7, is recruited to DNA in a damage-inducible and Rad9-dependent manner in *S. cerevisiae* [25]. In addition, in *S. pombe* polymerase κ physically interacts with 9-1-1, and its recruitment to chromatin is dependent on checkpoint activation [26]. These observations suggest a function of 9-1-1 in controlling TLS and possibly SHM in B cells. Most remarkably, a recent study in *S. cerevisiae* by Fu et al. indicated that DNA damage activates Rad6/Rad18 to ubiquitylate not only PCNA but also Rad17, the orthologue of mammalian Rad1 at a non-conserved lysine residue, K197 [27]. Furthermore, it was shown that Rad17 ubiquitylation controls phosphorylation of Rad53, the yeast Chk2 orthologue, a downstream component of the DNA damage response [27]. Strikingly, by solving the crystal structure of human 9-1-1, Doré et al. made the observation that the non-conserved Rad17^K197^ is not a topological equivalent of PCNA^K164^ [23]. In fact, Doré et al. revealed mammalian Rad1^K185^ as the only topological equivalent of PCNA^K164^ [23].

The facts that: 1) a topological equivalent of PCNA^K164^ exists in mammalian Rad1; 2) PCNA ubiquitylation by Rad6/Rad18 is selective for K164; and 3) that in yeast PCNA and 9-1-1 are both ubiquitylated in a DNA damage-inducible manner by Rad6/Rad18, prompted us to investigate whether the conserved mammalian Rad1^K185^ is not just a topological equivalent but also a functional counterpart of PCNA^K164^. To investigate the role of any PTMs of Rad1 in mammals, we introduced a K185R mutation in exon 4 of mouse *Rad1*. We found that the *Rad1* ^K185R^ mutation does not affect mammalian Chk1 activation, DNA damage survival, TLS function during SHM and class switch recombination (CSR) of Ig genes. These data are consistent with a recent report published by the Ulrich lab, suggesting that DNA damage-inducible ubiquitylation of 9-1-1 as observed by Fu et al. might not exist in yeast [28].

In addition, we simultaneously flanked exon 4 by LoxP-recombination sites. This strategy allows us to determine a putative role of Rad1^K185^ modification in mammalian DNA damage management and to inactivate *Rad1* conditionally in mammalian tissues. Cre-mediated deletion of exon 4 inactivates *Rad1*, providing an ideal model system to perform structure function analyses of Rad1 in a mammalian system.

## Materials and Methods

### Cloning of Rad1K185R targeting vector

The 5′ arm of homology (∼3 kbp) was amplified with a PmeI site at the 5′ end and an AscI site at the 3′ end (FWD: 5′-TTT TGT TTA AAC ACC AGA CTG GCT TCA AGT TCT TG-3′ and REV: 5′-TTT GGC GCG CCT CTT TAA AGA CAC CTG ATT CCA A-3′). The 3′ arm of homology (∼2.5 kbp) was amplified with a SbfI site at the 5′ end and a NotI site at the 3′ end (FWD: 5′-TTT CCT GCA GGG TAA CCA CAA AGC ATT TTA TA-3′ and REV: 5′-TTT GCG GCC GCT GTT TGG ATC CAC TAA ATG CCA TGC-3′). To generate a *Rad1* exon 4 containing the K185R mutation the 5′ portion of exon 4 was amplified using a natural HindIII site in the FWD primer 5′-GCA TGC TAG AAG CTT GGC AGA T-3′ and the mutagenic reverse primer: 5′- GCA CTG ACG TAC CTG AAA TAC GGC CGG TCA GGA GAC ACA GTG ATC T-3′. The 3′ portion of exon 4 was amplified using the mutagenic forward: 5′-AGA TCA CTG TGT CTC CTG ACC GGC CGT ATT TCA GGT ACG TCA GTG C-3′ and the reverse primer REV: 5′-TTT TTA ATT AAC TCA AGG TTG GAA AAT TAT GGA AT-3′ containing a PacI site at the 3′ end. To obtain the HindIII, PacI flanked K185R mutant exon4 of *Rad1*, the partial products were mixed and amplified using the FWD and the REV primer. All PCR products were amplified with Pfu polymerase (Promega) and subcloned in the TOPO blunt vector (Invitrogen) for sequencing (3730 DNA analyzer, Applied Biosystems). To generate the targeting vector, the fragments containing the 5′ arm of homology (AH), the 3′ of AH and the mutated *Rad1* exon 4 were cloned into the pFLEXIBLE targeting vector [29], using the indicated restriction sites.

### Generation of Rad1K185R mice and genotyping

**Figure 1:**
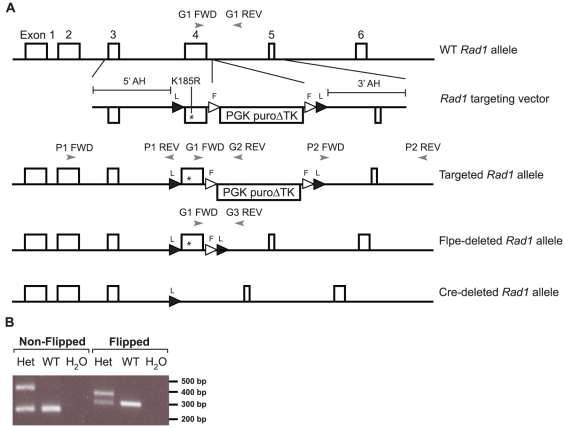
Targeting strategy and genotyping *Rad1* ^K185R^ mouse. A) Targeting strategy *Rad1* ^K185R^ mouse. LoxP recombination sites are represented by black triangles. Flpe recombination sites are represented by white triangles. PCR primers are represented by gray arrow heads. Please note that this figure is not drawn to scale. B) Genotyping PCRs for non-flipped (Primers G1 FWD, G1 REV and G2 REV) and flipped *Rad1* ^K185R^ mice (Primers G1 FWD and G3 REV).

E14 129/Ola embryonic stem cells were electroporated with NotI linearized *Rad1* ^K185R^ targeting vector. To screen for homologous recombination of the targeting vector in targeted ES cells, DNA was extracted from the ES cell clone and PCR primers specific for the proper integration of the targeting vector (5′AH: FWD: 5′-CCC CGG AGA TAG AGT CTA ACA TG-3′ (P1 FWD, Figure 1A); REV: 5′-TAG CAT ACA TTA TAC GAA GTT ATG GCG-3′ (P1 REV, Figure 1A) and 3′AH: FWD: 5′-GTA TGC TAT ACG AAG TTA TCC TGC AG-3′ (P2 FWD, Figure 1A); REV: 5′-GAG GGC TTC AGT AGC GAC AGC-3′ (P2 REV, Figure 1A)) were used. PCR cycle: 1) 94°C, 2 minutes; 2) 94°C, 30 seconds; 3) 60°C, 1 minute; 4) 72°C, 3 minutes; 5) 72°C, 10 minutes. Step 2 to 4 were repeated 34 times.

Homologous recombinant E14 129/Ola ES cell clones with a normal karyotype were injected into B6 blastocysts to obtain chimeric mice. To detect chimeric mice with a mutant *Rad1* allele, mice were genotyped with the following PCR primers: FWD: 5′-AGG TAC GTC AGT GCG ATT ACC CT-3′ (G1 FWD, Figure 1A); REV1: 5′-GTA GAA GGT GGC GCG AAG GGG-3′ (G1 REV, Figure 1A) and REV2: 5′-GTA GAT TAT GAG AAT CGG CTT CCA AC-3′ (G2 REV Figure 1A). Germline competent mice were crossed with the Flpe deleter strain (provided by S. Dymecki, Harvard Medical School, Boston, MA) to delete the selection cassette *in vivo* [30]. Genotyping of Flpe deleted *Rad1* ^K185R^ mice: FWD: G1 FWD (Figure 1A) and REV 5′-CCC TCA AGA TGT AAC CTC ATC TAC-3′ (G3 REV, Figure 1A).

All experiments were approved by an independent animal ethics committee of the Netherlands Cancer Institute (ID 8065) and executed according to national guidelines.

### Derivation of *Rad1* ^K185R^ mouse embryonic fibroblast cell lines

Mouse embryonic fibroblasts (MEFs) were derived from embryos at day 14.5 of gestation. MEFs were maintained in complete medium (IMDM, 8% FCS, 50 µM 2-mercapthoethanol, penicillin/streptomycin). Immortalization of MEF cell lines was established by lentiviral-mediated shRNAs targeting p53 [31].

### DNA damage survival

Naive splenic B cells from three mice per genotype were obtained by CD43 depletion using biotinylated anti CD43 (Clone S7, BD Biosciences), and the IMag system (BD Biosciences), as described by the manufacturer. For UV-C irradiation, 10^5^ B cells were irradiated (254 nm, UV Stratalinker® 2400, Stratagene) in 0.5 ml complete medium containing 50 µg/ml *E. Coli* LPS (055:B5, Sigma). For γ-irradiation, a ^137^Cs source was used. Following irradiation, cells were cultured in 1 ml complete medium and LPS. To determine DNA damage sensitivity, the survival of 10^5^ B cells grown in 1 ml complete medium and LPS in the continuous presence of different doses of cisplatin (CisPt) or methyl methanesulfonate (MMS) was determined after four days of culture. The number of viable (propidium iodine negative) B cells was determined by FACS. Data were analyzed using FlowJo 8.8.6 software.

### Isolation of germinal center B cells and mutation analysis

Germinal center (CD19+, PNA high, CD95+) B cells were sorted from Peyer's patches. Genomic DNA was extracted using proteinase K treatment and ethanol precipitation. The JH4 3′flanking intronic sequence of endogenous rearrangements of VHJ558 family members were amplified during 40 cycles of PCR using PFU Ultra polymerase (Stratagene). PCR products were purified using the QIAquick Gel Extraction kit (Qiagen) and cloned into the pCR-Blunt II TOPO vector (Invitrogen Life Technologies) and sequenced on a 3730 DNA analyzer (Applied Biosystems). Sequence alignment was performed on the first 300 bp starting from the intronic region using Seqman software (DNAStar). Calculations exclude non-mutated sequences, insertions, deletions, and SNPs. Clonally related sequences were counted only once. Statistical analysis was performed as described [17].

### Class switch recombination

Naive splenic B cells from three mice per genotype were obtained by CD43 depletion as described above. Purified B cells were cultured in complete medium containing LPS either in the presence or absence of 10% IL-4-containing supernatants generated from X63-m-IL-4 cell cultures [32]. Flow cytometric analysis of surface Ig expression was performed on day 4 of culture using goat anti mouse IgM-APC, IgG1-PE and IgG3-PE (Southern Biotech). Data were analyzed using FlowJo 8.8.6 software.

### Chk1 activation Western blotting

One day prior to UV irradiation wild type and *Rad1* ^K185R^ MEFs were seeded at 1.6*10^6^ cells per 15 cm dish in 20 ml complete medium. The next day, cells were washed with PBS and irradiated with 100 J/m^2^ UV-C (254 nm, UV Stratalinker® 2400, Stratagene) after removal of the PBS. Hereafter complete medium was added. 10, 40 and 70 minutes later cells were harvested by scraping the cells in cold PBS, centrifuged (500×g). After removal of the supernatant, cells were lysed in 200 µl ELB buffer (150 mM NaCl; 50 mM Hepes pH 7.5; 5 mM EDTA; 0.1% NP-40; protease inhibitors (Roche)) and incubated for 30 minutes on ice. Next, samples were centrifuged for 10 minutes at 20,800×g (4°C). The supernatant was transferred to a new tube and the protein concentration was measured using standard Bradford method. Western blotting was performed using standard protocols. NuPAGE 3-8% Tris-Acetate gels (Invitrogen) were used for protein separation. Antibodies used were: mouse anti-Chk1, 1∶1000 (sc-8408, Santa Cruz); rabbit anti-pChk1 S345, 1∶1000 (clone 133D3, Cell Signaling); mouse anti-Actin, 1∶10,000 (clone C4 (MAB1501R), Milipore).

## Results

### Generation of *Rad1* ^K185R^ mutant mice with a floxed exon 4

To test the possible role of Rad1^K185^ modifications in controlling mammalian DDT, we generated a mouse mutant with a site-specific *Rad1* ^K185R^ mutation in exon 4 of the *Rad1* locus (Figure 1A). Simultaneously, we also flanked this exon with LoxP recombination sites, which allows conditional inactivation of the *Rad1* ^K185R^ allele and functional analysis of Rad1 in higher eukaryotes. To identify homologous recombinants, we established a long range PCR strategy to detect homologous recombinant ES cells (primer sets P1 and P2, Figure 1A). To prevent possible detrimental effects of the selection cassette, *Rad1* ^K185R^ mice were crossed with the Flpe deleter strain to remove the selection cassette *in vivo* [30]. Mice homozygous for *Rad1* ^K185R^ were obtained by intercrossing heterozygous mice. Heterozygous and homozygous *Rad1* ^K185R^ mice were born at Mendelian ratios, indicating that the *Rad1* ^K185R^ mutation has no detrimental effect on mouse development (data not shown).

### Rad1^K185^ does not control Chk1 activation

**Figure 2:**
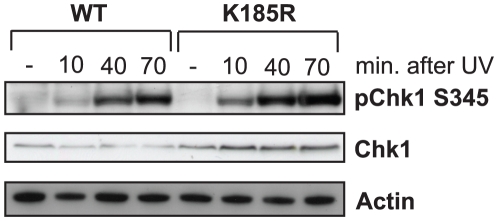
*Rad1* ^K185R^ MEFs have normal Chk1 activation. WT and *Rad1* ^K185R^ MEFs were irradiated with 100 J/m^2^ UV-C and harvested after 10, 40 and 70 minutes after irradiation. Subsequently, the Chk1 phosphorylation status at S345 (pChk1 S345) was investigated by Western blotting using pChk1 S345-specific antibodies. The results are representatives of two independent experiments.

Mammalian 9-1-1 has been implicated in the activation of the checkpoint kinase Chk1, a critical activation step for DDR [24]. For example, upon UV irradiation *Hus1*-deficient MEFs display significant lower levels of serine (S) 345 phosphorylated Chk1 (pChk1 S345) [33]. Moreover, Fu et al. have shown that PTM of 9-1-1 plays a role in DDR activation as well, as *rad17-K197R sgs1Δ* yeast cells also have an impaired DDR [27]. These observations led us to postulate that possible PTMs at Rad1^K185^ could also contribute to the activation of the mammalian DDR. As opposed to *Hus1*-deficient MEFs and *rad17-K197R sgs1Δ* yeast cells, *Rad1* ^K185R^ MEFs do not display impaired DDR activation after DNA damage as revealed by pChk1 S345 levels (Figure 2).

### *Rad1* ^K185R^ B cells display normal DNA damage sensitivity

**Figure 3:**
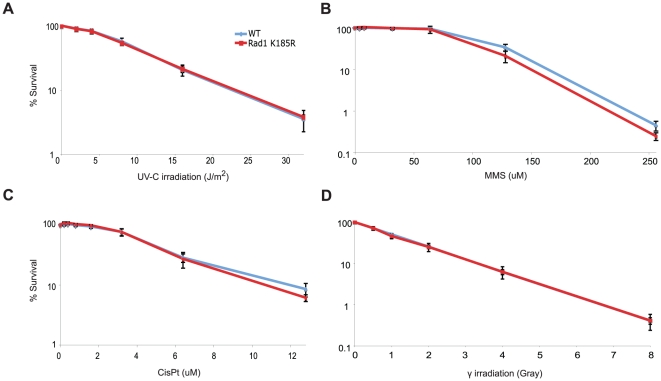
*Rad1* ^K185R^ B cells do not display sensitivity to various DNA damaging agents. WT (blue) and *Rad1* ^K185R^ (Red) B cells were stimulated with LPS and exposed to increasing amounts of UV-C (A), MMS (B), CisPt (C) and γ-irradiation (D). The percentage of survival is shown on the y-axis after four days of culture. Data represent the mean and SD of individual cultures (n = 3). The results are representatives of two independent experiments.

Modification of PCNA plays an important role in the regulation of DDT, as PCNA^K164R^ cells are extremely sensitive to various DNA damaging agents, primarily DNA damaging agents that cause replication blocking lesions [5]. Besides the importance of PCNA modification in DDT, 9-1-1 modification in yeast seems to play a role in DNA damage management as well [27]. Fu et al. showed that *rad17-K197R* yeast cells are sensitive to the alkylating agent methyl methanesulfonate (MMS) [27]. Hence, we determined the sensitivity of *Rad1* ^K185R^ B cells to replication blocking lesions such as induced by MMS, UV-C and CisPt. Moreover, as 9-1-1 was shown to be involved in the repair of DNA double strand breaks (DSBs) by means of homologous recombination [34], [35], we also investigated whether *Rad1* ^K185R^ B cells were more sensitive than WT cells to γ-irradiation. In contrast to *rad17-K197R* yeast cells, *Rad1* ^K185R^ B cells were as sensitive as WT B cells to MMS, as well as CisPt, UV-C and γ-irradiation (Figure 3).

### Rad1^K185^ is not involved in the regulation of SHM and class switch recombination (CSR)

**Figure 4:**
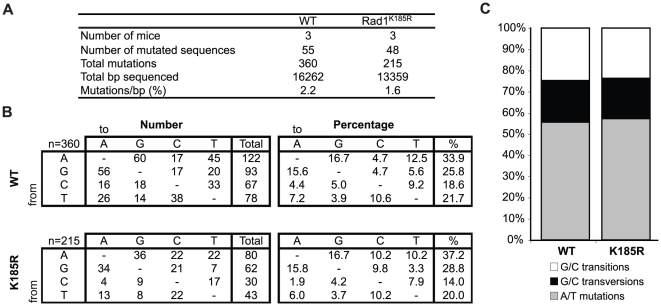
Normal SHM in R*ad1* ^K185R^ GC B cells. A) Mutated JH4 regions from WT and *Rad1* ^K185R^ GC B cells. B) *Rad1* ^K185R^ GC B cells display a normal nucleotide exchange pattern in hypermutated Ig genes. In the left panel, values are expressed as the total numbers of mutations. In the right panel, values are expressed as the percentage of total mutations. Chi square testing did not reveal any significant changes in the nucleotide exchange pattern (*p*<0.01). C) Relative contributions of A/T mutations, G/C transversions and G/C transitions in the different mouse strains. Values are expressed as the percentage of total mutations.

The majority of point mutations generated during SHM depend on TLS [11]. During non-SHM TLS, the TLS polymerases Rev1, polymerase η and κ need their ubiquitin binding motifs to efficiently interact with PCNA-Ub after DNA damage [6], [7], [8], [9]. However, during SHM only polymerases η and κ require PCNA-Ub for their recruitment. Polymerases η and κ are responsible for the generation 90% of all mutations at template A/T around the initial mismatch [16], [17], [18], [19], [36], [37]. Interestingly, TLS polymerases involved in G/C transversions, like Rev1, are not controlled by PCNA-Ub, suggesting that G/C transversions are regulated differently [16], [17]. Rev1 interacts with Rev7, and Rad9 can recruit Rev7 to the site of DNA damage [25]. Additionally, 9-1-1 was shown to physically interact with polymerase κ and that recruitment of polymerase κ to the chromatin was dependent on checkpoint activation [26]. Therefore, we postulated a role for Rad1^K185^-specific modification in SHM. However, unlike *PCNA* ^K164R^ B cells, *Rad1* ^K185R^ B cells are capable of undergoing normal SHM, as we observed no significant changes in the base exchange pattern of JH4 intronic sequences of germinal center B cells (Figure 4).

**Figure 5:**
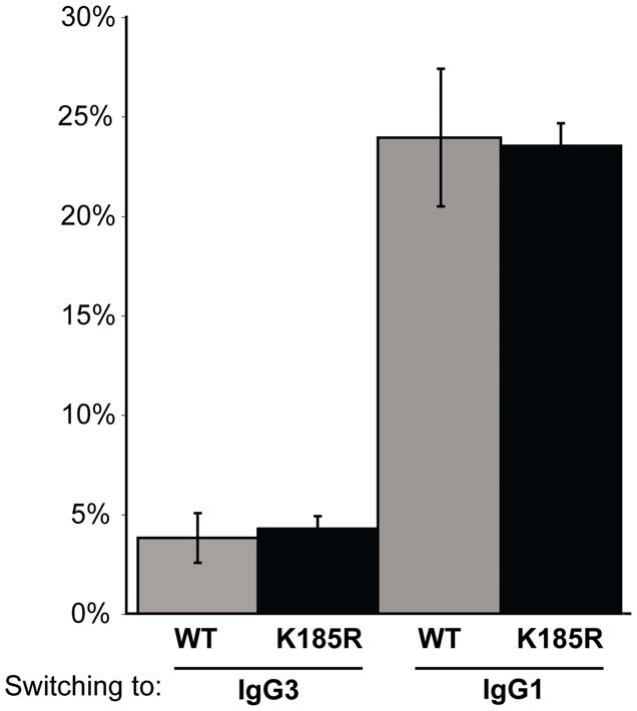
CSR is not altered in *Rad1* ^K185R^ B cells. WT (gray bars) and *Rad1* ^K185R^ (black bars) B cells were tested for their ability to switch to either IgG3 or IgG1 by stimulation with LPS or LPS and IL-4, respectively. Data represent the mean and SD of individual B cell cultures from three independent mice. The results are representatives of two independent experiments.

We also tested whether the *Rad1* ^K185R^ mutation had any effect on CSR in B cells (Figure 5). The *Rad1* ^K185R^ mutation does not affect *ex vivo* class switching of naive B cells to IgG3 or IgG1.

## Discussion

The DNA sliding clamps PCNA and 9-1-1 are critical docking stations for proteins involved in diverse processes such as replication, recombination, and DNA damage management. Site-specific PTM of these sliding clamps helps to coordinate the activation of specific pathways. Stalled replication forks activate the Ub-conjugase/ligase Rad6/Rad18 complex to mediate PCNA^K164^-specific ubiquitylation and subsequent stimulation of DDT. In this regard, the recent finding that in *S. cerevisiae* the same Rad6/Rad18 complex ubiquitylates Rad17, the yeast *Rad1* orthologue, at lysine residue 197 was quite intriguing [27]. However, Rad17^K197^ is not conserved and based on structural arguments unlikely to be a substrate of Rad6/Rad18 [23]. Yet, structural comparisons by Doré et al. did reveal a lysine residue (K185) in the Rad1 subunit of 9-1-1 that is indeed a topological equivalent of PCNA^K164^ [23].

To investigate whether PCNA^K164^ and Rad1^K185^ are not just topological equivalents, but also functional counterparts, we first tried to identify DNA damage-inducible Rad1 ubiquitylation in different mammalian cell lines. After extensive experimentation we were unable to observe any DNA damage-inducible PTMs, in particular ubiquitin modification of Rad1 (data not shown). As this approach proved unsuccessful for potentially a number of reasons, we took a genetic approach by introducing a *Rad1* ^K185R^ mutation in the mouse germline. Being aware of the fact, that equal topology does not necessarily imply equal functionality, we simultaneously flanked exon4 of *Rad1* with LoxP sites. This strategy allows conditional inactivation of endogenous *Rad1* and study structural variants of mammalian Rad1 in the absence of wild type Rad1.

Our data clearly demonstrate that any PTM at Rad1^K185^ does not play a role in DNA damage management, SHM or CSR. These studies are in line with recent observations made by the Ulrich lab [28]. Their results argued against a role of DNA damage-inducible and Rad17^K197^-specific ubiquitylation and the relevant phenotypes of the *rad17-K197R* yeast strain. Specifically, in this particular study the authors reported that modification of Rad17 is independent of: 1) DNA damage; 2) Rad6/Rad18; 3) the acceptor site Rad17^K197^; and 4) loading of the complex onto DNA, a prerequisite for PCNA^K164^ ubiquitylation [28]. Furthermore, the authors were unable to observe DNA damage sensitivity or defects in DNA damage checkpoint signaling in *rad17-K197R* yeast cells. Instead, they showed that all 9-1-1 subunits are (poly)ubiquitylated and that this modification likely directs proteasomal degradation [28].

Collectively, our data show that putative PTMs at Rad1^K185^ do not play a role in DNA damage management, which is in line with recent observations made in the Ulrich lab [28]. We conclude that mammalian Rad1^K185^ is a mere topological, but not a functional counterpart of PCNA^K164^.

Having flanked *Rad1* exon 4 with LoxP recombination sites allows a conditional inactivation of *Rad1* in mice and cell lines derived thereof. Upon deletion of exon 4, any alternative splicing gives rise to out-of-frame transcripts downstream of exon 3. As *Rad1* null embryos are not viable [38], our and equivalent systems of *Rad9* [39] and *Hus1* [40] will enable a detailed structure/function analysis of the mammalian 9-1-1 DNA sliding clamp in DNA damage management in future studies.
